# The sedentary behavior reduction in pregnancy intervention (SPRING) pilot and feasibility randomized trial

**DOI:** 10.1186/s12884-024-06474-3

**Published:** 2024-04-11

**Authors:** Bethany Barone Gibbs, Andrea C. Kozai, Shannon N. McAdoo, Kelliann D. Davis, Meghan B. Savidge, Joshua L. Paley, Alisse Hauspurg, Janet M. Catov

**Affiliations:** 1 Department of Epidemiology and Biostatistics, West Virginia University School of Public Health, PO Box 9190, 64 Medical Center Drive, Morgantown, WV 26506 USA; 2 Department of Epidemiology, University of Pittsburgh, Pittsburgh, PA USA; 3 Department of Health and Human Development, University of Pittsburgh, Pittsburgh, PA USA; 4 Department of Exercise Science, University of South Carolina, Columbia, SC USA; 5 Department of Obstetrics, Gynecology, and Reproductive Sciences, University of Pittsburgh, and Magee Women’s Research Institute, Pittsburgh, PA USA

**Keywords:** Thigh-worn accelerometer, Acceptability, Health coaching, Multi-level intervention

## Abstract

Pregnant individuals rarely achieve moderate-to-vigorous intensity physical activity recommendations.

**Purpose** The sedentary behavior reduction in pregnancy intervention (SPRING) pilot and feasibility randomized trial aimed to demonstrate feasibility, acceptability, and initial efficacy of a lower intensity intervention targeting reduced sedentary behavior and increased standing and steps.

**Methods** First trimester pregnant individuals at risk for high sedentary behavior and adverse pregnancy outcomes (APO) were randomized 2:1 to a multi-component sedentary behavior reduction intervention or no-contact control. Intervention components included biweekly remote health coaching, wearable activity monitor, height-adjustable workstation, and a private Facebook group. Evidence-based behavioral targets included sedentary time < 9 h/day, increasing standing by 2–3 h/day, and ≥ 7500 steps/day. Participants completed all-remote assessments (baseline, second trimester, third trimester) of sedentary behavior and activity (thigh-worn activPAL) along with exploratory pregnancy health outcomes abstracted from medical records. Intervention effects vs. control were evaluated using generalized mixed models and an intention-to-treat approach. Intervention participants also provided feedback on perceived benefits and acceptability.

**Results** Participants (34 intervention; 17 control) had mean age 32 years, were 83% White, with mean pre-pregnancy BMI 28 kg/m^2^. Retention was high (90% and 83% at second and third trimester follow-up visits). Intervention participants decreased sedentary time (-0.84 h/day, *p* = 0.019) and increased standing (+0.77 h/day, *p* = 0.003), but did not increase steps/day (+710, *p* = 0.257) compared to controls. Intervention participants reported many perceived benefits and identified the wearable, height-adjustable workstation, and behavioral lessons as most useful.

**Conclusion** For pregnant individuals at risk for high sedentary behavior and APOs, a sedentary behavior reduction intervention is feasible, acceptable, and may offer a viable alternative to more intense physical activity recommendations during pregnancy. Further testing in a fully powered clinical trial is warranted.

**Trial registration** NCT05093842 on clinicaltrials.gov

**Supplementary Information:**

The online version contains supplementary material available at 10.1186/s12884-024-06474-3.

## Introduction

Adverse pregnancy outcomes (APOs), such as hypertensive disorders of pregnancy (HDP) and gestational diabetes (GDM), have been increasing in prevalence in the U.S. in recent decades [1, 2]. For example, a 2023 U.S. Preventative Task Force report found that HDP incidence has doubled since 1993 [3]. These trends are alarming since APOs pose immediate threats to the health of the pregnant mother and her offspring, and experiencing an APO is now recognized as an important risk factor for underlying and future cardiovascular disease (CVD) [4, 5]. Interventions that prevent APOs offer immediate and possibly lasting intergenerational benefits. However, few evidence-based approaches for preventing APOs during pregnancy are available [6]. Participating in moderate-to-vigorous intensity physical activity does have an established benefit, with studies of exercise interventions conferring a risk reduction of ~40% for HDP and GDM [7, 8]. Yet, only an estimated 1 in 4 pregnant individuals meet physical activity guidelines [9]. The low levels of physical activity likely reflect a combination of barriers to physical activity experienced by the general population (e.g., lack of time, enjoyment, or access) overlaid upon barriers specific to pregnancy (e.g., fatigue, medical restriction, or concern for the baby) [10]. More feasible and effective lifestyle approaches to reduce APO risk are needed.

Reducing sedentary behavior (SED) by increasing standing and light intensity activity during pregnancy is a strategy that is distinct from and perhaps more feasible than higher intensity physical activity given pregnancy-specific barriers. SED is defined as low-intensity behavior, while awake, that occurs in a seated, reclined, or lying posture [11]. Accumulating evidence suggests that high levels of SED especially when accumulated in prolonged bouts are a risk factor for CVD and other health risks in non-pregnant adults, even after taking moderate-to-vigorous intensity physical activity into consideration [12, 13]. Though few studies have investigated associations between SED and pregnancy health outcomes [14], a small cohort study from our group [15] recently identified that pregnant individuals with the highest vs. lowest levels of SED across trimesters (approximately 11 vs. 8 h/day as measured by a thigh-worn accelerometer) had significantly elevated risk of a composite measure of APOs (OR = 6.76, 95% confidence interval: 1.20, 38.14). Moreover, individuals in the medium SED group (approximately 9 h/day) did not have excess APO risk. Having a medium or high level of time spent standing and a medium or high number of steps/day while pregnant was similarly associated with lower APO risk, suggesting these were each advantageous SED replacement behaviors. Of interest, objectively measured moderate-to-vigorous physical activity was not associated with APO risk in this cohort, lending more support for a strategy of reducing SED by increasing standing and light intensity moving. However, limited SED reduction-focused interventions have been developed and rigorously tested in pregnant individuals.

In response to this research gap, we conducted the Sedentary Behavior Reduction in Pregnancy Intervention (SPRING) pilot and feasibility study. The goals of the SPRING study were to develop and pilot a SED reduction intervention among pregnant individuals at risk for high levels of SED and for APOs. The primary aim of the study was to evaluate the effect of the intervention on daily activity patterns including durations of SED (primary outcome), standing, and stepping, as well as total steps/day. A secondary aim was to assess the feasibility and acceptability of the intervention and assessment methods. Lastly, we explored preliminary effects of the intervention on pregnancy health outcomes including blood pressure (BP), heart rate, glucose measures, gestational weight gain, and APOs.

## Methods

### Study design and setting

SPRING was a pilot and feasibility randomized clinical trial conducted in the greater Pittsburgh, PA (United States) area between September 2021 and June 2023. The SPRING study enrolled participants who were in their first trimester of pregnancy and had risk factors for both high SED and APOs. After completion of baseline assessments, participants were randomized 2:1 into parallel arms: either a multi-component SED reduction intervention or a control group. Intervention participants received health coaching by videoconference every 2 weeks through up to 38 weeks gestation along with a height-adjustable workstation, wrist-worn activity monitor, and membership in a private social media group. Participants completed follow-up assessments in the second and third trimesters, and maternal-fetal outcomes were abstracted from medical charts following delivery. All assessment and intervention procedures were conducted remotely. A detailed protocol has been published previously [16]. An overview of the assessment and intervention schedule is provided in Table 1.

**Table 1:**
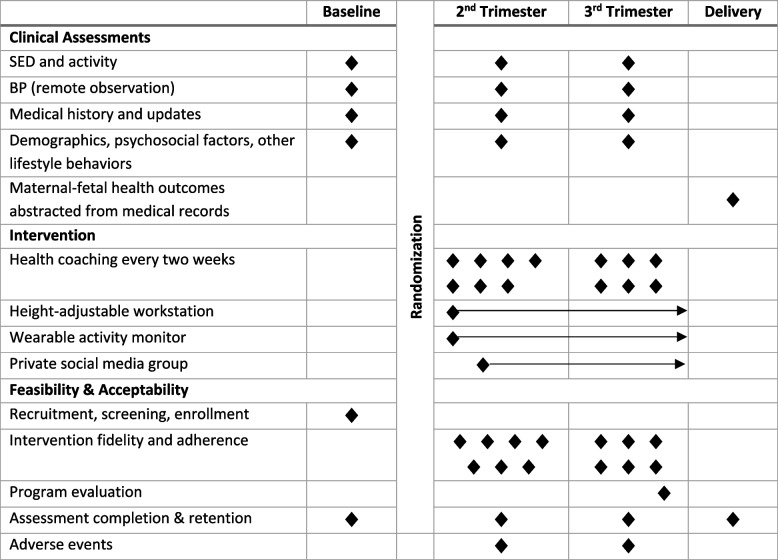
SPRING study schedule of assessments and intervention

This manuscript reports on the primary, secondary, and exploratory outcomes of the study. All research procedures were approved by the University of Pittsburgh Human Research Protection Office. The study was submitted for registration on clincialtrials.gov (NCT05093842) on September 23, 2021 and first posted on October 26, 2023.

### Participants and randomization

Participants were recruited from several sources, including the University of Pittsburgh Clinical and Translational Sciences Institute’s Research Registry (Pitt + Me), mass emails sent to University of Pittsburgh employees, direct messaging through the electronic health record to potentially eligible pregnant patients that attended prenatal visits in the study physician’s maternal fetal medicine clinic, and fliers placed in the Magee-Womens Hospital of the University of Pittsburgh. These methods directed participants to complete a REDCap® survey that assessed initial eligibility and shared contact information with the study coordinator. Individuals who continued to be eligible and interested were then scheduled for an orientation and consent visit by videoconference between their 10^0^ and 12^6^ weeks of pregnancy. At this visit, a trained study personnel began by conducting informed e-consent followed by a final assessment of study eligibility through a detailed medical and pregnancy history interview.

Inclusion criteria for the SPRING Study were: 1) gestational age between 10^0^ and 12^6^ at the time of baseline assessment; 2) at risk for high SED defined as meeting at least one of the following criteria: i) primarily sitting full-time desk job (≥ 30 h/week), ii) primarily sitting, part-time desk job (< 30 h/week) and reports sitting at least ½ of the time while not working; iii) does not work and reports sitting at least ¾ of the time; or iv) reports < 6000 steps/day from a wearable activity monitor; 3) has at least one risk factor for APO: i) nulliparity, ii) history of APO, iii) pre-pregnancy BMI ≥ 30 kg/m^2^, or iv) age ≥ 35 years old; and 4) planned to deliver at the University of Pittsburgh facility or willing to provide consent for medical record release of prenatal care and birth records. Individuals were excluded from participating in SPRING if any of the following were true: 1) young (< 18 years) or advanced (> 45 years) maternal age; 2) chronic hypertension defined as resting BP ≥ 140/90 mmHg or using antihypertensive medications at the time of screening; 3) pregestational type 1 or type 2 diabetes; 4) contraindication to exercise due to a serious medical condition; 5) severe mobility limitation defined as inability to walk two blocks or climb a flight of stairs; 6) unable to obtain a signed permission form from a prenatal care provider to participate in the intervention; 7) participation in another health-related intervention that could affect SPRING Study outcomes; or 8) plans to travel or other reasons that would limit ability to fully participate in the study protocol.

Participants deemed eligible during the orientation screening were then scheduled to complete a virtual baseline assessment visit that included measurement of BP and heart rate with remote observation, questionnaires that measured demographic, psychosocial factors, and other lifestyle behaviors, and assessment of SED and physical activity with a thigh-worn activPAL accelerometer for 1 week that was then returned by mail (see Assessments). At the same time, the project coordinator worked with the participant to obtain signed permission to participate from the participant’s prenatal care provider. Participants that successfully completed the baseline assessment, including mail return of the activPAL and prenatal care provider permission, were then cleared for randomization.

Randomization was conducted using the sealed envelope method with a 2:1 intervention:control ratio. This ratio with twice as many intervention participants was selected to provide additional data on the intervention’s feasibility and acceptability as well as enhance recruitment [17]. Blinded study personnel generated a set of sequentially numbered, sealed envelopes containing randomly ordered numbers in blocks of six. When a participant completed all baseline procedures, the blinded study coordinator alerted the randomization team (principal investigator and interventionist) who then opened the next sequential envelope and recorded the randomization assignment in a secured database. Next, the principal investigator conducted a phone call with each participant to provide them with their randomization assignment, describe next steps (either to connect to the interventionist or to enter the no-contact control group), and reinforce the importance of completing future assessments and maintaining blinding with the assessment staff regarding the randomized group assignment.

### Intervention

The multi-component SPRING intervention was designed by study investigators and intervention personnel. We adapted our previously successful SED reduction interventions in non-pregnant populations [18–20] with consideration of data from our cohort study of pregnant individuals to inform SED and activity targets [15, 21] and participant attitudes, barriers, and facilitators of SED and activity during pregnancy [22, 23]. An overview of the intervention components and schedule is provided in Table 1 with greater detail previously published [16]. Intervention components were selected to encourage SED reduction across socioecological levels and included health coaching by videoconference (individual/interpersonal level) [24–26], a participant-selected height-adjustable workstation (environmental level) [27], use of a wearable device to self-monitor activity breaks and steps (individual level) [28], and membership in a private social media (Facebook) group (interpersonal level) [29]. Virtual health coaching by a trained interventionist began at approximately 14 weeks of gestation and occurred every 2 weeks through delivery or 38 weeks of gestation, whichever came first.

SPRING’s evidence-based behavioral targets were to reduce SED to < 9 h/day by increasing standing by 2–3 h/day (with an overall goal of ≥ 4 h/day) and steps to ≥ 7,500 per day [15]. Six behavioral lessons with goal setting and designed to last 30–45 min alternated with up to seven goal setting check-ins that were designed to last 10–15 min [26, 30]. Lesson topics included education and review of baseline (pre-intervention) objective SED and activity data [24, 25], social support [29], stimulus control and environmental reengineering [31], progress review including review of objective SED and activity data from the second assessment visit [25, 32], motivation [33, 34], and relapse prevention [30]. The health coaches used a motivational interviewing-informed approach at each contact to review self-reported standing and movement breaks as well as recent activity data shared from the wearable, address barriers, and facilitate participant-led goal adjustment [26, 35]. At each intervention contact, participants were queried regarding new contraindications to exercise [36], and goals were revised with the consultation of the study physician if necessary.

Prior to the first intervention lesson, the interventionist consulted with the participant to provide a height-adjustable workstation that would allow the participant to complete some typically seated activities in a standing posture. Our research group has used several height-adjustable workstations in SED-reduction interventions, and we therefore allowed the participant to select one of our lab-approved devices that would be most appropriate for their lifestyle (e.g., desk job, not employed but have a home computer). Examples of devices commonly provided were desktop devices (e.g., Humanscale QuickStand Eco®, Ergotron Mini Z®) or stand-alone workstations (e.g., Ergotron LearnFit®, FlexiSpot Standard standing desk).

Also prior to the first intervention lesson, participants were engaged to select a wearable device to self-monitor steps and activity breaks. Based on our previous studies where participants disliked discontinuing use of an Apple Watch® for our study-provided fitbit®, we allowed SPRING participants to choose one of three options: 1) use of an existing Apple Watch® by sharing fitness data with the interventionist (*n* = 9); 2) use of an existing fitbit® by sharing account credentials with the interventionist (*n* = 2); or 3) receive a new, programmed fitbit Luxe® from the SPRING Study (*n* = 22). Both fitbit® and Apple Watch® can enable self-monitoring of daily steps and movement breaks, and this flexible approach allowed participants to continue use of the other functionalities of their wearable when applicable.

The final component of the intervention sought to engage social support through one of the virtual behavioral lessons that was delivered to a small group (2–4 intervention participants) followed by an invitation from the interventionist to join a study-facilitated private social media group on Facebook. Participants remained in the Facebook group until they delivered their child. SPRING interventionists posted twice weekly on the Facebook group with the goals to educate, engage, and entertain participants. Participants were not required to engage or post in the group but were encouraged to do so.

### Assessments

Virtual assessment visits were completed at baseline (10^0^ to 12^6^ weeks gestation), in the second trimester (20^0^ to 22^6^ weeks gestation), and in the third trimester (32^0^ to 34^6^ weeks gestation) by blinded study personnel. Following delivery, maternal-fetal outcomes from prenatal visits, labor and delivery, and birth were abstracted from medical records (see Table 1).

#### Demographics and other questionnaires

At baseline, participants completed a standardized questionnaire with demographic information about their age, race/ethnicity, and employment status.

#### Medical history and adverse events

At baseline, study personnel conducted a medical history interview to obtain information on reproductive history, medical conditions, and medication use to describe the population and determine eligibility. At subsequent assessment visits, a blinded study personnel systematically asked participants to report any changes in medications and medical conditions. New or worsening medical conditions were classified as adverse events. Detailed information on adverse events, including severity and possible relation to assessment or intervention procedures, was obtained and reviewed by the principal investigator.

#### SED and physical activity

SED (primary outcome) and physical activity were measured at each study assessment using a thigh-worn activPAL accelerometer (activPAL3, PALtechnologies, Glasgow, Scotland) that was mailed to the participant prior to the remote assessment. Participants were verbally instructed and provided with detailed written instructions to wear the device affixed to their anterior thigh with medical tape for 24 h × 8 days, with removal only for swimming activities [37]. Proper placement was verified by study personnel during the remote assessment. Participants were asked to complete a concurrent wear diary that reported time in bed, wake and sleep times, and non-wear periods. After wear, participants returned the monitor and diary by prepaid mailer.

Using our laboratory’s standard processing procedures, activPAL data were downloaded, exported as event-type files using the PALTehcnologies software, and a diary-informed cleaning approach was used to classify waking, sleep, and non-wear times [15, 38]. When possible, participants were asked to rewear the monitor in cases of malfunction or incorrect wear. In rare cases when a diary was incomplete or lost but the activity monitor was returned, participants were asked to provide typical bed and wake times, and these were used with our standard approach to score the data. For individuals with at least 5 valid days of wear [39], durations of waking time spent in SED (i.e., total), SED30 (SED accumulated in bouts of ≥ 30 min), SED60 (SED accumulated in bouts of ≥ 60 min), standing, and stepping as well as steps per day and waking wear time were averaged across valid days. We also estimated time spent in higher intensity physical activity from 1-min epoch data by averaging minutes with a rate of ≥ 75 steps/minute (stepping75) or ≥ 100 steps/minute (stepping100) across valid days, a method that we have previously shown to accurately estimate moderate-to-vigorous intensity physical activity during pregnancy [40].

#### BP

Given our remote assessment protocol, all participants were asked whether they had been given a UA-611 BP monitor (A&D Medical, Ann Arbor, MI) by their prenatal care provider. Providing this validated [41] monitor to patients was a common practice during our recruitment period by the Magee Womens Hospital of the University of Pittsburgh. If not, participants were mailed a validated [41] BP 7250 monitor (Omron Health Inc., Lake Forest, IL) to keep.

Using a consistent monitor within-subject, virtual assessment of BP occurred at baseline and both follow-up visits. The protocol began with a verbal confirmation of abstention from caffeine and nicotine for the previous hour. Next, participants were instructed to secure the BP cuff on the left arm and sit quietly for 5 min with the left arm supported at heart level, back supported, legs uncrossed, and feet supported on the floor or a footrest. After the observed rest, the assessor asked the participant to initiate the BP measurement on the oscillometric monitor that was facing toward the video camera and away from the participant. The assessor asked the participant to avoid looking at the readings and recorded the BP and heart rate reading in the REDCap database. This was repeated two more times, with a 1-min rest interval in between readings. Participants were provided with all three readings after the measurement and advised to consult a healthcare professional if the average systolic BP was ≥ 140 or diastolic BP was ≥ 90 mmHg. The final two readings were averaged for analysis.

#### Maternal health outcomes from medical records

Clinical outcomes abstracted from medical records included office BP from prenatal visits, screening glucose from a 50g screen, pre-pregnancy weight and weight at the time of delivery to estimate gestational weight gain, and APOs. Three BPs were abstracted to align with the end of the first trimester (closest to but not exceeding 13 weeks gestation), the second trimester (closest to but not exceeding the 28 weeks gestation), and the third trimester (final prenatal visit prior to delivery). APOs were classified using guidelines and standard definitions for HDP (gestational hypertension or preeclampsia) [6], GDM [42], preterm birth (< 37 weeks gestation), and small-for-gestational-age (SGA, < 10th percentile sex-specific birthweight for gestational age) [43]. All outcomes were abstracted by trained research personnel and then reviewed for accuracy by a study investigator (B.B.G.). APOs were additionally reviewed by the study maternal-fetal medicine physician (A.H.) for accuracy.

#### Feasibility, acceptability, and fidelity

Recruitment feasibility and acceptability were measured by the frequency of screening contacts by recruitment method used, enrollment and reasons for ineligibility, and characteristics of the enrolled participants. Retention feasibility was measured as the frequency of assessment completion with a benchmark of 80% for follow-up visits and recording of adverse events.

Intervention fidelity was evaluated as the frequency of delivery for intervention lessons and components, with a benchmark of 85%. In addition, frequency of receipt and enactment across intervention components along with duration of intervention contacts were recorded. Lastly, intervention acceptability was assessed by an investigator-developed program evaluation questionnaire that asked participants to report perceived benefits/unfavorable effects, usefulness of individual components and lesson topics, alignment with expectations, and other preferences and suggestions.

### Statistical analysis

We calculated a required sample size of 42 participants (28 intervention, 18 control) to be 90% powered to detect a 1-h difference in SED between group, assuming a standard deviation of 56 min [15], a 2:1 randomization ratio, and a two-sided α = 0.05 (as previously reported [16]). We inflated the required sample size by 20% to account for attrition (*n* = 53) but stopped recruitment at *n* = 51 as *n* = 42 participants had already completed at least one follow-up assessment visit.

All analyses were conducted using Stata version 16.0. Feasibility and acceptability data were summarized descriptively using a CONSORT diagram, proportions, and means (SD). Demographic and clinical characteristics of the participants at baseline were compared across randomized groups using means (SD) and independent *t* tests or frequencies (%) and Chi-square tests, as appropriate. Baseline characteristics of participants who completed study visits vs. those missing outcomes at follow-up visits were similarly compared.

Primary analyses used an intention-to-treat (ITT) approach. SED and activity variables were compared across randomized groups at follow-up visits using mixed models, adjusting for baseline levels, and with multiple imputation of missing data (10 imputations using Stata *mi impute* commands). Two participants were removed from this analysis due to miscarriage after randomization. Sensitivity analyses repeated comparisons across intervention groups using observed data only. Similar ITT analyses were used to test differences in BP and heart rate across randomized groups at follow-up timepoints. Generalized linear models were used to calculate differences in the glucose screen, gestational weight gain, and the odds of APOs across randomized groups where appropriate, again with imputation for missing data. In the case of small sample sizes for rare APOs (*n* < 5), Fisher’s exact tests were used to evaluate group differences. For all pregnancy clinical or health outcomes, two additional participants were removed from analyses due to twin pregnancies that could impact these outcomes.

## Results

### Feasibility of recruitment and retention

Recruitment was conducted between September 2021 and September 2022. Of 140 screening forms, the greatest proportion were referred by the University of Pittsburgh’s Pitt + Me research registry (49%), followed by maternal-fetal medicine patient referral (27%), and e-mail advertisements sent to the University of Pittsburgh community (19%). Personal referrals, internet searches, and flyers provided smaller yields (1–2% each).

**Fig. 1:**
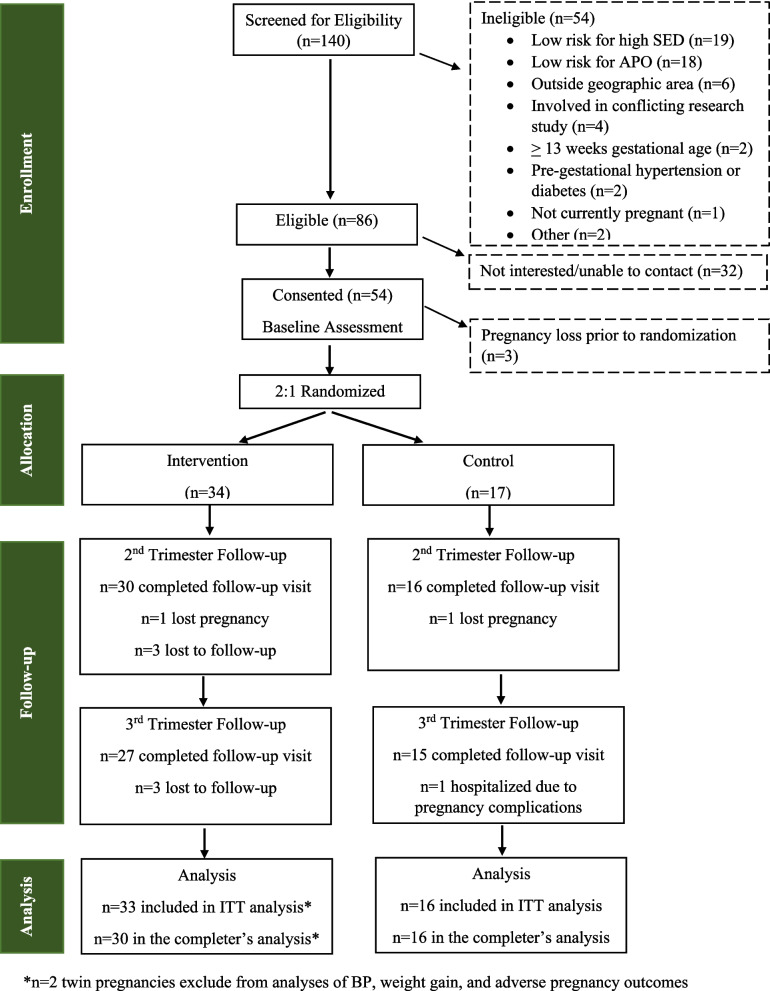
SPRING study CONSORT diagram

The CONSORT Diagram (Fig. 1) details study progression from screening through follow-up. Of 140 screening forms received, 86 participants (61%) were initially eligible. Reasons for ineligibility were most commonly not meeting criteria for risk of high SED or high risk for APO. Of those eligible after initial screening (*n* = 86), 51 (59%) progressed to randomization. Of those who did not enroll in the study, most discontinued screening due to lack of interest, though three discontinued due to pregnancy loss.

Characteristics of participants by randomized group are compared in Table 2. No statistically significant differences in demographics or clinical characteristics were observed by group, though the proportion of participants reporting White race was meaningfully higher in control (100%) vs. intervention (73.5%). Retention suffered from additional losses of pregnancy (*n* = 2) and losses to follow-up (*n* = 7), but still exceeded the 80% benchmark with 90% follow-up at the second trimester follow-up visit and 83% for the third trimester follow-up visit.

**Table 2:** Baseline characteristics of SPRING participants

	**Intervention (*n* = 34)**	**Control (*n* = 17)**	***P*-value**
**Demographics**			
Age, mean	31.7 (4.7)	32.5 (3.6)	0.559
Race, n(%)			0.075
White	25 (74)	17 (100)	
Black	6 (18)	0 (0)	
Other	3 (9)	0 (0)	
Ethnicity, n(%)			0.610
Non-Hispanic	33 (97)	16 (94)	
Hispanic	1 (3)	1 (6)	
Employment, n(%)			0.481
Full-time	22(65)	13 (76)	
Part-time	3 (9)	2 (12)	
Not currently employed	9 (27)	2 (12)	
**Pregnancy Characteristics and History**			
Gestational age at baseline, weeks	11.8 (0.8)	11.8 (0.6)	0.881
Pre-pregnancy BMI, kg/m^2^	28.1 (9.7)	28.1 (7.7)	0.978
Parity			0.480
Nulliparous	20 (59)	7 (41)	
1	9 (26)	6 (35)	
2 or more	5 (15)	4 (24)	
History of APO (among previously pregnant)			0.770
No Previous History	5 (36)	3 (30)	
Yes Previous APO	9 (64)	7 (70)	

*Abbreviations*: *APO* Adverse pregnancy outcome, *BMI* Body mass index

### Effect of the intervention on primary outcomes: SED and activity across pregnancy

SED and activity from activPAL monitoring were captured in 46 and 42 participants at the second and third trimester follow-up visits, respectively. Participants who did not provide follow-up activPAL data were younger (*p* < 0.05) but otherwise like those with data (Supplemental Table 1).

SED and activity across pregnancy are compared across randomized groups in Table 3. In our primary ITT analyses, intervention participants averaged significantly less total SED (-0.84 h/day, *p* = 0.019) and less SED accumulated in prolonged bouts (SED30: -0.99 h/day, *p* = 0.014; SED60: -1.05 h/day, *p* = 0.008) as compared to controls in the second and third trimesters of pregnancy. Intervention participants also had significantly more standing (+0.77 h/day, *p* = 0.003) than controls, though time spent stepping overall, higher intensity stepping (reflective of moderate-to-vigorous intensity physical activity), and steps per day did not differ by randomized group. Results were unchanged in a sensitivity analysis that only included observed data (Supplemental Table 2).

**Table 3:** SED and activity across pregnancy by randomized group (ITT, *n* = 49)

	1^st^ trimester (baseline)	2^nd^ trimester (follow-up)	3^rd^ trimester (follow-up)	β_intervention_ (SE)	*P*-value
**SED**					
SED total, hr/day					
Intervention	10.42 (0.28)	9.69 (0.29)	9.62 (0.29)	**-0.84 (0.36)**	**0.019**
Control	10.52 (0.40)	10.66 (0.37)	10.38 (0.34)	ref.	
SED30, hr/day					
Intervention	6.34 (0.36)	5.30 (0.37)	5.20 (0.42)	**-0.99 (0.40)**	**0.014**
Control	5.98 (0.61)	5.96 (0.62)	5.91 (0.61)	ref.	
SED60, hr/day					
Intervention	3.82 (0.39)	2.56 (0.32)	2.44 (0.41)	**-1.05 (0.39)**	**0.008**
Control	3.26 (0.59)	3.30 (0.61)	2.95 (0.55)	ref.	
**Activity**					
Standing, hr/day					
Intervention	2.79 (0.20)	3.43 (0.26)	3.81 (0.26)	**0.77 (0.26)**	**0.003**
Control	2.74 (0.29)	2.80 (0.28)	2.81 (0.31)	ref.	
Stepping, hr/day					
Intervention	1.14 (0.09)	1.50 (0.11)	1.48 (0.13)	0.20 (0.13)	0.132
Control	1.38 (0.18)	1.46 (0.17)	1.41 (0.19)	ref.	
Stepping75, min/day					
Intervention	16.17 (2.84)	22.84 (3.83)	21.16 (3.79)	2.61 (3.78)	0.491
Control	18.47 (3.87)	21.40 (5.05)	21.33 (4.18)	ref.	
Stepping100, min/day					
Intervention	9.76 (2.22)	12.82 (2.45)	10.18 (2.39)	-0.79 (2.41)	0.745
Control	10.55 (3.02)	12.10 (3.86)	13.13 (3.18)	ref.	
Steps per day					
Intervention	5224 (502)	6929 (553)	6777 (656)	710 (627)	0.257
Control	6268 (895)	6775 (913)	6479 (919)	ref.	

Visit-specific values are reported as mean (SE) and β_intervention_ corresponds to the difference between the intervention and control group at both follow-up visits, adjusting for baseline levels, and with multiple imputation, from a linear mixed model. The ITT analysis excludes *n* = 2 participants who lost their pregnancies after randomization

*Abbreviations*: *hr/day* Hours per day, *ITT* Intention-to-treat, *SE* Standard error, *SED* Sedentary behavior, *SED30* Sedentary behavior accumulated in bouts of at least 30 min, *SED60* Sedentary behavior accumulated in bouts of at least 60 min, *stepping75* Daily duration of time spent stepping at a rate of at least 75 per minute, *stepping100* Daily duration of time spent stepping at a rate of least 100 per minute

### Effect of the intervention on exploratory outcomes: BP, heart rate, gestational weight gain, and APOs

The intervention did not influence BP or heart rate measured during remote study visits or BP abstracted from the medical chart. The difference in BP between randomized groups (β_intervention_) ranged from -2.7 mmHg to 1.2 mmHg, with all *p* > 0.2 (see Supplemental Table 3).

**Fig. 2:**
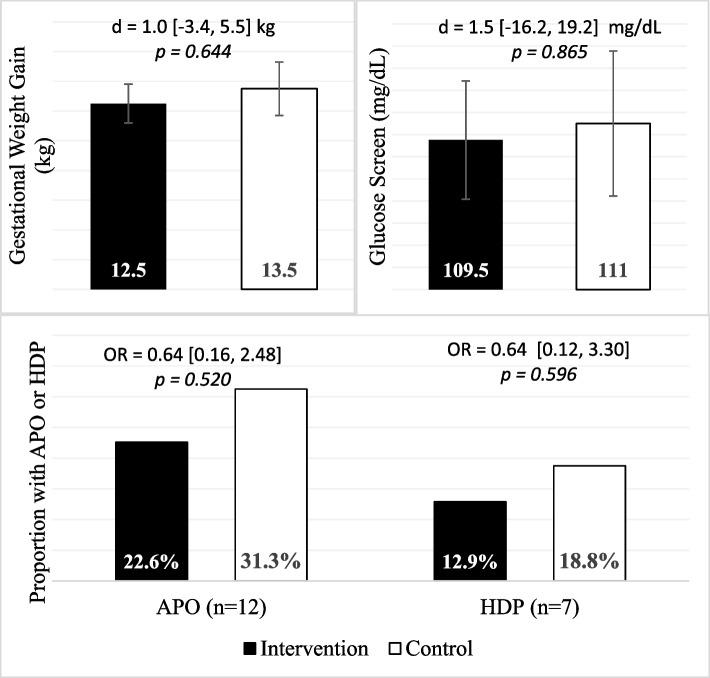
APO, gestational weight gain, and screening glucose by randomized group (ITT, *n* = 47). Differences (d) and odds ratios (OR) were calculated from generalized linear models and compare outcomes in the intervention group vs. the control group. 95% confidence intervals are reported in the brackets following estimates. Abbreviations: APO, adverse pregnancy outcomes (composite); HDP, hypertensive disorders of pregnancy

Figure 2 displays exploratory outcomes measured at only one time point including gestational weight gain, glucose screen, odds of APO, and odds of HDP. Of note, HDP was the only APO with sufficient incidence to consider separately. No statistically significant differences across randomized groups were observed. Preterm births, GDM, and SGA were infrequent (*n* = 2 each, see Supplemental Table 4).

### Fidelity and acceptability of the intervention

Intervention fidelity, feasibility, and acceptability are summarized in Table 4. Intervention fidelity was above the 85% benchmark for delivery/receipt of intervention lessons (88%), delivery/receipt of the height-adjustable workstation and activity monitor (all ≥ 98%), and for the interventionist sending the Facebook group friend request (94%). Just below the 85% benchmark were average delivery/receipt of intervention check-ins at 83% and Facebook group invitation acceptance by the participant at 81%. Average enactment was only above benchmarks for wearing and self-monitoring of steps and activity breaks with the activity monitor (> 96%) but was lower for self-monitoring of standing time (65%) and visiting the Facebook group at least weekly (17%).

**Table 4:** Intervention fidelity and acceptability of the SPRING intervention

**Fidelity** (*n* = 33)			
**Component**	**Delivery**	**Receipt**	**Enactment**
Intervention lessons (6 lessons)	Completed^a^: 88% (79–94%)	Duration^a^: 39.9 ± 6.6 min	n/a
Intervention check-ins (7 contacts)	Completed^a^: 83% (68–94%)	Duration^a^: 13.7 ± 4.7 min	n/a
Height adjustable^b^ workstation	Provided: 100%	Working^a^: 100% (97–100%)	Self-monitoring standing^a^: 65% (17–83%) Self-reported standing^a^: 2.3 ± 1.3 h/day
Activity monitor^b^	Provided: 100%	Working^a^: 98 (90–100%)	Wearing regularly^a^: 97% (90–100%) Self-monitoring movement breaks and steps^a^: 97% (92–100%) Objective movement breaks^a^: 7.8 ± 3.7 breaks/day Objective steps^a^: 6521 ± 2571 steps/day
Facebook group	Interventionist sent request: 94%	Participant accepted request: 81%	Reported visiting at least weekly: 17%
**Acceptability** (*n* = 24)			
**Usefulness of components** *Range: 0 (not at all) to 3 (very much)* Activity monitor: 2.6 ± 0.6 Height adjustable workstation: 2.5 ± 0.8 Intervention check-ins: 1.9 ± 0.6 Intervention lessons: 1.8 ± 0.6 Facebook group: 0.6 ± 0.6		**Lesson ratings** *Range: 0 (not helpful) to 2 (very helpful)* Lesson 1 Education: 1.8 ± 0.4 Lesson 2 Social support: 1.0 ± 0.7 Lesson 3 Get to know your cues: 1.6 ± 0.5 Lesson 4 Progress report: 1.5 ± 0.5 Lesson 5 Motivation: 1.5 ± 0.6 Lesson 6 Lapses don’t have to be collapses: 1.5 ± 0.5	
**General ratings** *Agree or strongly agree, unless otherwise noted* Intervention had a positive effect on my pregnancy: 96% Increased understanding of the health risks of sitting during pregnancy: 79% Expectations were met: 88% Intervention asked too much of my time: 13% Format preference: 58% (no change), 13% (no group meeting), 38% (more group meetings) Will continue changes made during the intervention: 46% (probably will) and 54% (definitely will)			

Quantitative data presented as mean ± SD or % (range of %)

^a^Assessed across multiple intervention contacts. Overall averages with standard deviations or ranges of visit-specific proportions are presented

^b^Percentage reported among participants completing the visit

From the program evaluation completed after the last intervention lesson (Table 4, acceptability), participants found the activity monitor and height adjustable workstation to be the most useful components of the intervention (2.5–2.6 of 3 possible points), the intervention lessons and check-ins to be moderately useful (1.5–1.6 of 3 points), and the Facebook group to be least useful (0.6 of 3 points). Of the six lessons, the initial education lesson content was rated as most helpful (1.8 of 2 points) and the social support lesson was rated as least helpful (1.0 of 2 points). Two frequent themes emerged from responses to an optional, open-ended question asking for ways we could improve the intervention. First, six participants suggested improving the social aspect of the intervention by offering more opportunities to interact with other group members (e.g., more group meetings, online group fitness classes, group walks, competitions) or using a different social media platform. Second, four participants mentioned that check-ins with the interventionist were too frequent, especially at the end of the study.

**Fig. 3:**
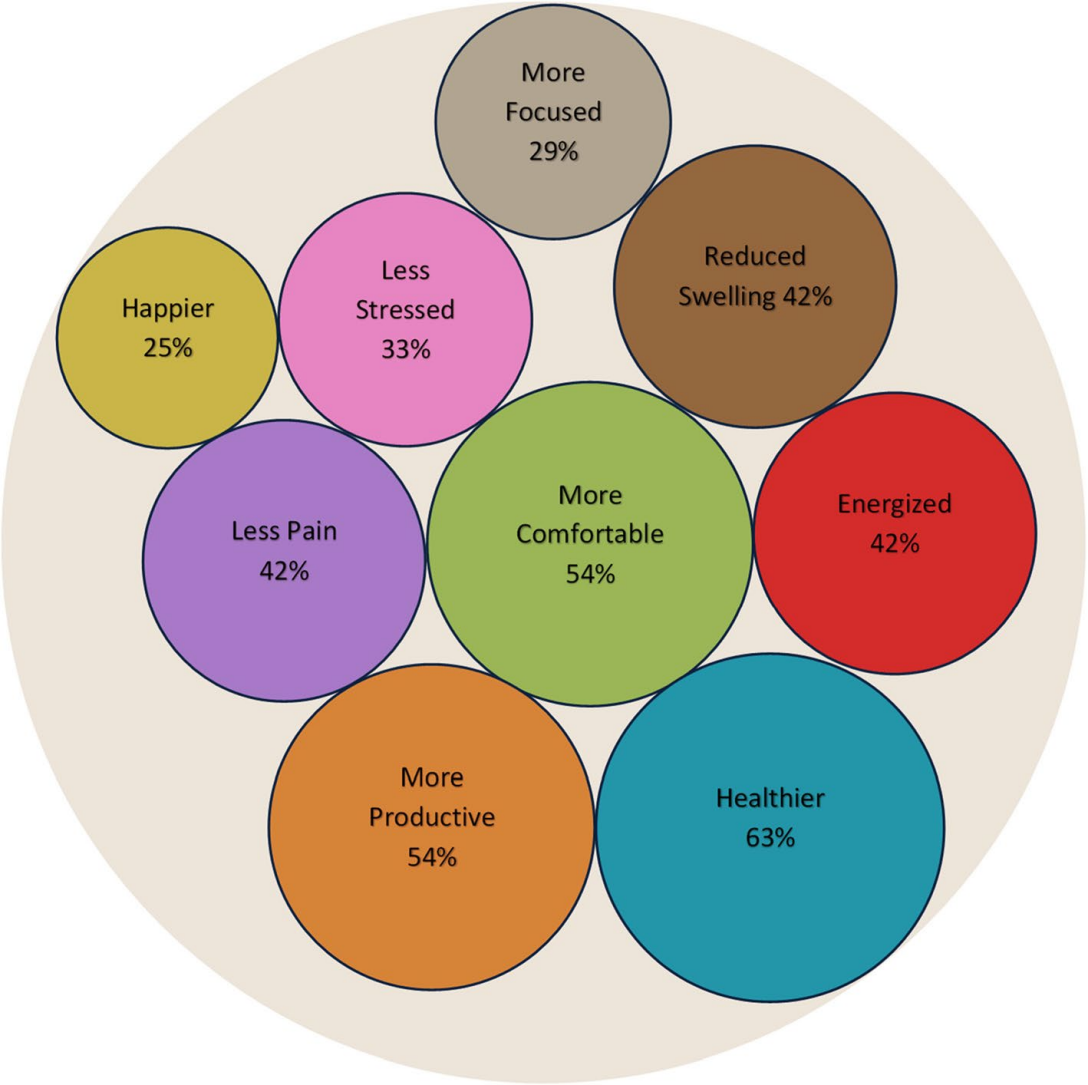
Proportion of participants reporting benefits from the SPRING intervention (*n* = 24)

A high proportion of participants agreed with the statement that the intervention had a positive effect on their pregnancy (96%) (Table 4). When provided a list of possible benefits, most participants reported that they felt healthier (63%), more comfortable (54%), and more productive (54%) because of the intervention. Nearly half (42%) reported feeling energized, reduced swelling, and having less pain. Other less frequently reported benefits and frequencies are shown in Fig. 3. Most stated they increased their knowledge of SED (79%) and that expectations were met (88%), while few felt the intervention took too much time (13%) (Table 4). Regarding format, a majority recommended no change (58%), but 38% suggested more group meetings. All participants said they probably (46%) or definitely (56%) would continue changes made during the intervention.

### Adverse events

Adverse events from systematic questioning at assessment visits were rare, with four (12%) in the intervention group and five (31%) in the control group (Supplemental Table 5). There were no severe adverse events, and none of the adverse events were classified as possibly or definitely related to the research assessments or intervention.

## Discussion

The SPRING pilot and feasibility study sought to test the effects of a behavioral intervention on SED and activity, to evaluate feasibility and acceptability, and to explore preliminary effects of the intervention on maternal health outcomes among pregnant individuals who had elevated risk for SED and APOs. SPRING was effective at reducing SED across pregnancy, most notably with time spent in prolonged bouts of SED being reduced by more than an hour each day as compared to the control group. Our intervention also resulted in significant increases in standing time compared to controls, though increases in stepping time and steps per day failed to reach statistical significance. Future interventions may need to provide more specific supports to promote greater steps per day. Our recruitment and retention efforts were successful, meeting benchmarks, while the fidelity and acceptability assessments provided important information on commonly perceived benefits and more favorable (behavioral lessons, height-adjustable workstation, wearable) and less favorable aspects (Facebook group, behavioral check-ins) of the intervention. Lastly, exploratory analyses of preliminary effects of the intervention on maternal health outcomes were all nonsignificant but typically in a favorable direction, with no evidence of adverse events associated with the intervention. Altogether, these data provide preliminary support for future testing of a SED-reduction intervention on maternal health outcomes among pregnant individuals with high SED and risk factors for APOs in a larger randomized controlled trial.

Strengths of the SPRING Study include the parallel randomized controlled trial design, especially across pregnancy where health behaviors and outcomes are labile. Our trial was rigorously conducted according to methods that were pre-registered (clinicaltrials.gov: NCT05093842) and reported in a published protocol [16]. A further strength was our novel adaptation of SED reduction strategies from successful interventions in general populations to a pregnant population; we did this using information about the determinants of SED from our previous cohort study along with evidence-based SED and activity targets associated with better pregnancy health outcomes. We had high retention and best practice device-based measures of SED and activity. Lastly, we carefully evaluated feasibility, acceptability, and fidelity of our intervention to inform an improved future intervention. Weaknesses of the study included the small sample, the selected population who all reported high SED at enrollment and had risk factors for APOs, and the limited racial/ethnic diversity. These limit the generalizability of our findings to general pregnant populations.

We are unaware of other randomized controlled trials with a primary focus on SED-reduction during pregnancy, though other studies have used similar approaches to increase physical activity (e.g., all remote, health coaching, wearable activity monitor) [44–46]. Most similar was the INSPiRE study [44], a single-arm intervention (*n* = 34) conducted in Iowa that used remote health coaching and a fitbit to increase physical activity (i.e., steps) and reduce SED. INSPiRE also utilized a thigh-worn activPAL to measure changes in steps and SED. Compared to baseline (and not a control group as in our study), INSPiRE participants significantly increased steps (+1,715 per day), increased standing time (+2%), and decreased SED (-4%). Our study, that had a primary goal of reducing SED and added a height-adjustable workstation to the intervention, realized larger within-intervention group reductions in SED (approximately -6 to -10% within group) and had greater increases in standing (approximately +7%). Increases in steps in our study were similar within the intervention group (approximately +1600 steps per day), though the change was not different when compared to a control group. This highlights the potential for activity patterns to change across pregnancy even without intervention and the importance of comparison to a control group. These data suggest that an intervention specifically targeting SED reduction, including the provision of a height-adjustable workstation, may have a greater influence on SED reduction and standing during pregnancy than behavioral counselling and a wearable activity monitor alone. These data are also consistent with systematic reviews of SED reduction interventions in non-pregnant populations that find interventions focused on SED reduction rather than increasing physical activity are more effective at reducing SED and that interventions including environmental-level components (e.g., height-adjustable workstations) are most effective and typically reduce SED by ~ 1 h per day [47, 48]. This notion is reinforced in the recent Danish FitMum Study (*n* = 220) that found fitbit-measured SED across pregnancy was not impacted by either a supervised exercise intervention or a motivational counselling on physical activity intervention (neither of which specifically targeted SED) [49].

Our results are also comparable to a 2022 meta-analysis that summarized the effectiveness of 18 randomized physical activity intervention trials with device-measured physical activity in pregnant populations [50]. Pooled estimates found that pregnant individuals receiving a physical activity intervention achieved 435 more steps per day and gained 0.69 kg less weight during gestation, compared to controls. The authors of this meta-analysis also concluded that future interventions should focus on total physical activity (e.g. steps per day) and not just moderate-to-vigorous intensity physical activity as a strategy to overcome pregnancy-specific barriers to being active such as fatigue. As suggested by these authors, the SPRING intervention focused on reducing SED and increasing standing and all-day activity and achieved nonsignificant but potentially clinically meaningful increases in steps per day (+710, *p* = 0.257) and reductions in gestational weight gain (-1.0 kg, *p* = 0.644). These observed effect sizes, though exploratory and thus underpowered to draw strong conclusions, were more favorable than the pooled estimates of changes in steps and gestational weight gain from this meta-analysis of physical activity interventions [50]. Similarly exploratory, comparing intervention vs. control in the SPRING intervention, observed rates of APOs (36% lower, *p* = 0.520) and specifically HDP (36% lower, *p* = 0.526) are comparable to pooled estimates from a meta-analysis of 106 studies that found exercise-only interventions are estimated to reduce HDP and GDM by 38–41% [7].

Taken together, our multi-component intervention that focused on reducing SED and increasing standing and steps during pregnancy effectively reduced SED, increased standing, and appears promising with respect to adherence, feasibility, favorable risk profile, and preliminary evidence suggesting similar pregnancy health benefits comparable to more intense physical activity interventions. Given the significant barriers and low population-level adherence to physical activity recommendations during pregnancy despite the substantial health benefits, the behavioral targets in SPRING may offer a palatable alternative for realizing the benefits of being active during pregnancy. Since our strategy notably did not increase higher intensity physical activity as is currently recommended, further testing is needed prior to updating pregnancy physical activity guidelines. Rigorous evaluation of our strategy to decrease SED through increasing standing and steps during pregnancy is needed, and ideally with direct comparison to current pregnancy physical activity guidelines which recommend 150 min per week of moderate-to-vigorous intensity aerobic physical activity [24, 51]. Future research should also evaluate the comparative effects of SED reduction through increasing standing and steps on other health outcomes important to pregnant populations including sleep, psychosocial well-being, and musculoskeletal health.

## Conclusion

The SPRING pilot and feasibility intervention significantly decreased SED and increased standing during pregnancy. Coupled with high feasibility and acceptability and potentially favorable effects on exploratory outcomes, testing of a refined SED intervention in a fully powered randomized clinical trial to rigorously evaluate this alternative approach to improve pregnancy health in those with high SED and risk factors for APOs is warranted.

### Supplementary Information

**Supplementary Material 1.**

## Data Availability

No datasets were generated or analysed during the current study.
