# Supplementary material for: The sedentary behavior reduction in pregnancy intervention (SPRING) pilot and feasibility randomized trial
Source: BMC Pregnancy Childbirth. 2024 Apr 11;24:261. doi: 10.1186/s12884-024-06474-3 (PMC11007988; doi:10.1186/s12884-024-06474-3)
Supplement: Supplementary file 1 — Supplementary Material 1. [file 12884_2024_6474_MOESM1_ESM.docx]

**Online Supplemental Materials**

**Online Supplemental Table 1. Characteristics of Participants with Complete or Missing activPAL Data (Visit 2 and or Visit 3)**

|  | **Observed V2** | **Missing V2** | **p-value** | **Observed V3** | **Missing V3** | **p-value** |
| --- | --- | --- | --- | --- | --- | --- |
| **Demographics** | **n=46** | **n=3** |  | **n=42** | **n=7** |  |
| Age, mean | 32.3 (3.9) | 25.3 (5.7) | **0.005** | 32.3 (4.0) | 28.6 (5.0) | **0.045** |
| Race, n (%)  White  Black  Other | 37 (80)  6 (13)  3 (7) | 3 (100)  0  0 | 1.000 | 34 (81)  5 (12)  3 (7) | 6 (86)  1 (14)  0 (0) | 1.000 |
| Ethnicity, n (%)  Non-Hispanic  Hispanic | 44 (96)  2 (4) | 3(100)  0 (0) | 0.880 | 40 (95)  2 (5) | 7 (100)  0 (0) | 0.732 |
| Employment, n (%)  Full-time  Part-time  Not currently employed | 32 (70)  4 (9)  10 (23) | 2(67)  0(17)  1(33) | 1.000 | 29 (69)  4 (10)  9 (21) | 5 (71)  0 (0)  2 (29) | 1.000 |
| **Pregnancy History** |  |  |  |  |  |  |
| Gestational Age at Baseline, weeks | 11.8 (0.7) | 12.3 (0.5) | 0.255 | 11.8 (0.7) | 12.0 (0.7) | 0.4342 |
| Pre-pregnancy BMI, kg/m^2^ | 27.9 (8.7) | 30.4 (14.5) | 0.652 | 27.7 (8.6) | 30.3 (11.5) | 0.485 |
| Parity  Nulliparous  1  2 or more | 24 (52)  15 (33)  7 (15) | 2 (67)  0 (0)  1 (33) | 0.418 | 23 (55)  13 (31)  6 (14) | 3 (43)  2 (29)  2 (29) | 0.652 |
| History of APO (among previously pregnant)  No Previous History  Yes Previous APO | 8 (36)  14 (64) | 0 (0)  1 (100) | 0.652 | 6 (32)  13 (68) | 2 (50)  2 (50) | 0.435 |

Abbreviations: APO, adverse pregnancy outcome; BMI, body mass index

**Supplemental Table 2. SED and Activity Across Pregnancy by Randomized Group (observed data analysis)**

|  |  | 1^st^ trimester  (baseline)  n=48 | 2^nd^ trimester  (follow-up)  n=46 | 3^rd^ trimester  (follow-up)  n=42 | β_intervention_ (SE) | p-value |
| --- | --- | --- | --- | --- | --- | --- |
| **SED** | |  |  |  |  | |
| Total, hr/day | Intervention | 10.4 (0.3) | 9.6 (0.3) | 9.5 (0.3) | **-0.99 (0.31)** | **0.001** |
|  | Control | 10.5 (0.4) | 10.7 (0.4) | 10.4 (0.3) | ref |  |
| SB30, hr/day | Intervention | 6.3 (0.4) | 5.2 (0.3) | 5.0 (0.3) | **-1.07 (0.37)** | **0.004** |
|  | Control | 5.9 (0.6) | 6.0 (0.6) | 5.9 (0.6) | ref |  |
| SB60, hr/day | Intervention | 3.7 (0.4) | 2.4 (0.3) | 2.2 (0.3) | **-1.11 (0.36)** | **0.002** |
|  | Control | 3.3 (0.6) | 3.3 (0.6) | 3.0 (0.5) | ref |  |
| **Activity** | | | | | | |
| Standing, hr/day | Intervention | 2.8 (0.2) | 3.5 (0.3) | 3.9 (0.2) | **0.77 (0.23)** | **0.001** |
|  | Control | 2.7 (0.3) | 2.8 (0.3) | 2.7 (0.3) | ref |  |
| Stepping, hr/day | Intervention | 1.2 (0.1) | 1.5 (0.1) | 1.6 (0.1) | 0.20 (0.13) | 0.115 |
|  | Control | 1.4 (0.2) | 1.5 (0.2) | 1.4 (0.2) | ref |  |
| Steps75, min/day | Intervention | 16.3 (2.9) | 24.5 (3.5) | 23.6 (3.8) | 3.11 (3.59) | 0.387 |
|  | Control | 18.5 (3.9) | 21.4 (5.0) | 20.3 (4.1) | ref |  |
| Steps100, min/day | Intervention | 10.0 (2.1) | 13.4 (2.4) | 11.6 (2.1) | -0.48 (2.28) | 0.832 |
|  | Control | 10.5 (3.0) | 12.1 (3.9) | 13.0 (3.1) | ref |  |
| Steps per day | Intervention | 5368 (477) | 7145 (531) | 7144 (567) | 889 (602) | 0.140 |
|  | Control | 6268 (895) | 6775 (913) | 6398 (905) | ref |  |

Visit-specific values are reported as mean (SE) and β_intervention_ corresponds to the difference between the intervention and control group at both follow-up visits, adjusting for baseline levels, and with multiple imputation, from a linear mixed model.

Abbreviations: hr/day, hours per day; ITT, intention-to-treat; SE, standard error; SED, sedentary behavior; SED30, sedentary behavior accumulated in bouts of at least 30 minutes; SED60, sedentary behavior accumulated in bouts of at least 60 minutes; stepping75, daily duration of time spent stepping at a rate of at least 75 per minute; stepping100, daily duration of time spent stepping at a rate of least 100 per minute

**Supplemental Table 3. Blood Pressure, Heart Rate, and Weight across Pregnancy by Randomized Group (ITT, n=47)**

|  |  | 1^st^ trimester  (baseline) | 2^nd^ trimester  (follow-up) | 3^rd^ trimester  (follow-up) | β_intervention_ (SE) | P-value |
| --- | --- | --- | --- | --- | --- | --- |
| **Study Visits** | | | | | | |
| Follow-up window (gestational age) |  | 10-12 weeks | 20-22 weeks | 32-34 weeks |  |  |
| SBP, mmHg | Intervention | 103.1 (1.9) | 102.3 (1.7) | 107.9 (2.4) | 0.7 (2.4) | 0.763 |
|  | Control | 108.3 (2.2) | 105.2 (2.3) | 109.2 (1.9) | ref. |  |
| DBP, mmHg | Intervention | 67.9 (1.4) | 66.6 (1.4) | 71.8 (1.6) | 1.2 (1.7) | 0.485 |
|  | Control | 72.6 (2.4) | 69.1 (2.3) | 72.4 (2.1) | ref. |  |
| HR, bpm | Intervention | 76.8 (2.0) | 80.0 (1.8) | 85.6 (2.1) | 1.6 (2.5) | 0.516 |
|  | Control | 80.3 (3.0) | 80.6 (3.3) | 85.3 (3.9) | ref. |  |
| **Medical Record Abstraction** | | | | | | |
| Gestational age parameter |  | ≤13 weeks | ≤28 weeks | Last visit prior to delivery |  |  |
| Gestational age at measurement, weeks | Intervention | 10.5 (0.3) | 26.0 (0.2) | 38.4 (0.4) |  |  |
|  | Control | 10.8 (0.4) | 25.1 (0.3) | 38.1 (0.4) |  |  |
| SBP, mmHg | Intervention | 112.5 (2.3) | 110.3 (1.5) | 115.7 (1.7) | -2.7 (2.3) | 0.242 |
|  | Control | 113.9(3.0) | 114.2 (2.7) | 117.6 (2.5) | ref. |  |
| DBP, mmHg | Intervention | 69.4 (1.3) | 67.3 (1.1) | 71.4 (1.4) | -1.3 (2.1) | 0.530 |
|  | Control | 73.3 (2.1) | 69.9 (2.0) | 73.8 (2.7) | ref. |  |

Visit-specific values are reported as mean (SE) and β_intervention_ corresponds to the difference between the intervention and control group at both follow-up visits, adjusting for baseline levels, and with multiple imputation, from a linear mixed model. The ITT analysis excludes n=2 participants who lost their pregnancies after randomization and n=2 participants with twin pregnancies due to possible influence on these outcomes.

Abbreviations: bpm, beats per minute; DBP, diastolic blood pressure; HR, heart rate; ITT, intention-to-treat; mmHg, millimeters of mercury; ref., reference group; SE, standard error; SBP, systolic blood pressure

**Supplemental Table 4. Outcomes from Medical Record Review (ITT, n=47)**

|  |  | Mean (SE) |  |  |
| --- | --- | --- | --- | --- |
| Gestational Weight Gain, kg | Intervention | 12.5 (1.3) |  | 0.644 |
|  | Control | 13.5 (1.8) |  |  |
| Glucose Screen, mg/dL^*^ | Intervention | 109.5 (5.35) |  | 0.865 |
|  | Control | 111 (6.55) |  |  |
|  |  | N events (%) | OR (95% CI) | p-value |
| APO (composite) | Intervention | 7 (22.6) | 0.64  (0.17, 2.48) | 0.520 |
|  | Control | 5 (31.5) | 1.0 (Ref.) |  |
| HDP | Intervention | 4 (12.9) | 0.64  (0.12, 3.30) | 0.596 |
|  | Control | 3 (18.8) | 1.0 (Ref.) |  |
| Preeclampsia | Intervention | 2 (6.5) |  | 0.541^†^ |
|  | Control | 0 (0) |  |  |
| Gestation Hypertension | Intervention | 2 (6.5) |  | 0.320^†^ |
|  | Control | 3 (18.8) |  |  |
| Preterm Birth | Intervention | 1 (3.2) |  | 1.000^†^ |
|  | Control | 1 (6.3) |  |  |
| Gestational Diabetes | Intervention | 1 (3.2) |  | 1.000^†^ |
|  | Control | 1 (6.3) |  |  |
| Small-for-gestational-age | Intervention | 2 (6.5) |  | 0.541^†^ |
|  | Control | 0 (0) |  |  |

*4 missing from medical chart due to involvement in another research study

^†^calculated using Fisher’s exact test due to small number of events

Abbreviations: APO, adverse pregnancy outcomes; HDP, hypertensive disorders of pregnancy

**Supplemental Table 5. Adverse Events by Group**

|  | Intervention group (n=33) | Control group (n=16) |
| --- | --- | --- |
| Adverse events, n (%) |  |  |
| Mild | 2 (6) | 1 (6) |
| Moderate | 2 (6) | 4 (25) |
| Severe | 0 (0) | 0 (0) |
| Total | 4 (12) | 5 (31) |
| Adverse events possibly or definitely related to assessment, n (%) | 0 (0) | 0 (0) |
| Adverse events possibly or definitely related to intervention, n (%) | 0 (0) | 0 (0) |
| Expected adverse events | 0 (0) | 1 (6) |
| Serious adverse events | 0 (0) | 0 (0) |
